# Randomized controlled trial of resistance exercise and brain aging clocks

**DOI:** 10.1007/s11357-026-02141-x

**Published:** 2026-02-10

**Authors:** Raul Gonzalez-Gomez, Naiara Demnitz, Carlos Coronel, Anne Theil Gates, Michael Kjaer, Hartwig R. Siebner, Carl-Johan Boraxbekk, Agustin M. Ibanez

**Affiliations:** 1 Latin American Brain Health Institute (BrainLat), Universidad Adolfo Ibañez, Santiago de Chile, Chile; 2 Neurology and Neurosurgery Department, Montreal Neurological Institute, Montreal, Canada; 3 Danish Research Centre for Magnetic Resonance, Department of Radiology and Nuclear Medicine, Copenhagen University Hospital - Amager and Hvidovre, Hvidovre, Copenhagen, Denmark; 4 Trinity College Dublin, The University of Dublin, Dublin, Ireland; 5 School of Medicine, Global Brain Health Institute (GBHI), Trinity College Dublin, Dublin, Ireland; 6 Institute of Sports Medicine Copenhagen (ISMC), Bispebjerg Hospital, University of Copenhagen, Copenhagen, Denmark; 7 Institute for Clinical Medicine, Faculty of Medical and Health Sciences, Copenhagen University, Copenhagen, Denmark; 8 Department of Neurology, Copenhagen University Hospital - Bispebjerg and Frederiksberg, Copenhagen, Denmark; 9 Department of Clinical Medicine, Faculty of Health and Medical Sciences, University of Copenhagen, Copenhagen, Denmark; 10 Cognitive Neuroscience Center, Universidad de San Andrés, Buenos Aires, Argentina; 11 Department of Biophysics, School of Medicine, Istanbul Medipol University, Istanbul, Türkiye; 12 Barcelonaβeta Brain Research Center (BBRC), Pasqual Maragall Foundation, Barcelona, Spain

**Keywords:** Exercise, Muscle strength, Brain health, Brain clocks, Functional connectivity

## Abstract

Exercise improves cognition, mental wellbeing, and protects against neurodegeneration. However, most prior neuroscience studies have focused on localized brain changes without quantifying their impact on brain ageing. To quantify the effect of resistance training on brain health using longitudinal assessments. Using resting-state functional magnetic resonance imaging (rs-fMRI) data from 2,433 healthy adults, we trained models to predict brain age and applied them to 309 participants from the Live Active Successful Aging (LISA) randomized trial. Participants in this trial were assigned to one of three groups: heavy-resistance training, moderate-intensity training, or a non-exercise control group. They underwent repeated rs-fMRI and physical fitness assessments at baseline, with follow-up assessments at 1 and 2 years. First, we examined changes in local connectivity between groups. Second, we assessed the impact of resistance training on brain ageing using brain clock models trained on the independent dataset of 2,433 adults. Local analyses revealed increased prefrontal functional connectivity following heavy training, while moderate- and heavy-resistance training significantly reduced brain age (–1.4 to –2.3 years, p_FDR_ < 0.05). These effects emerged at the whole-brain level, rather than within isolated networks such as the default mode, motor, or cerebellar systems. These findings suggest a hierarchical organization of brain aging, driven by distributed network-level changes and expressed through focal regional patterns. Resistance exercise training decelerates brain ageing, as indexed by brain clocks, reinforcing its role as a preventive strategy for brain health.

**Supplementary Information:**

The online version contains supplementary material available at 10.1007/s11357-026-02141-x.

## Introduction

Exercise has been consistently linked to improvements in cognitive function [1], brain volume—particularly within the hippocampus [2–4]—as well as functional connectivity [5]. However, findings across studies remain mixed [6], and substantial individual differences in response to training have been observed [7, 8]. Traditionally, the effects of exercise on the brain have been quantified through neuroimaging outcomes such as regional brain volumes, cortical thickness, and connectivity measures [9–13]. While these markers have demonstrated relevant associations, they often capture changes in isolated brain regions and do not provide a direct measure of delayed or accelerated aging effects. Brain clock models have recently emerged as integrative biomarkers of brain health [14–18]. These models estimate an individual’s brain age from multimodal neuroimaging, and the difference from chronological age (brain age gap) is interpreted as an index of brain health status [14–16]. Lifestyle habits have been shown to exert protective effects, delaying brain aging [19–21]. Thus, brain clocks may provide a powerful tool for evaluating whether exercise interventions decelerate brain aging.

Despite the evidence linking exercise to improvements in brain health, few studies have applied computational models such as brain clocks. Prior research has left gaps in understanding the global impact of exercise on brain aging. Findings are mixed, and the predominance of short-term or cross-sectional designs further limits insights into long-term trajectories [11–13]. To date, most studies have concentrated on the effects of cardiovascular training, whereas the neural correlates of resistance training remain comparatively underexplored. Recent evidence, however, suggests that while strength training may have limited effects on brain structure [22], resistance training could modulate functional brain activity and enhance working memory performance [23]. However, the extent to which resistance exercise translates into measurable changes in brain aging trajectories remains poorly understood, especially regarding brain clocks and functional connectivity.

The present study aimed to quantify the impact of resistance training on brain health through longitudinal assessments over a one-year intervention. We included a subsample of 309 older adults from the controlled LISA trial, who had been randomized into three groups: heavy resistance training (HRT), moderate-intensity resistance training (MIT), and a non-exercise control group. Resting-state functional magnetic resonance imaging (rs-fMRI) was employed to characterize changes in functional networks. First, we examined local connectivity changes between groups, replicating the design of previous studies [9–13]. Second, we assessed the impact of resistance training on brain aging using brain clock models trained on an independent dataset of 2,433 subjects to test whether the effect was global or driven by specific networks (e.g., motor network). We hypothesized that exercise training, particularly HRT, would slow brain aging trajectories compared with non-exercise controls, as reflected in both mass-univariate analyses and brain clock outcomes. Furthermore, we expected that improvements in physical function over the course of the intervention would translate into more favorable trajectories of brain aging.

## Materials and methods

### Participants

A total of 2742 healthy individuals were included, of which 2433 came from external databases used to train the brain clock models (training sample). The remaining 309 participants, representing the study sample, were obtained from the Live Active Successful Aging (LISA) trial [24], a randomized, supervised, one-year strength-training intervention designed to examine the effects of physical exercise on muscle strength, cognitive function, and overall well-being in older adults approaching retirement age. Implemented at the Institute of Sports Medicine Copenhagen (ISMC) and the Danish Research Center for Magnetic Resonance (DRCMR), Denmark, the LISA study included assessments of magnetic resonance imaging, physical function, and demographic characteristics. The external datasets relied on resources widely used in previous brain health research.

### Ethics and consent to participate declarations

All procedures were conducted in accordance with principles outlined in the Declaration of Helsinki, and with approval from the Ethical Committees of the Capital Region of Denmark (No. H-3–2014-017) and the Danish Data Protection Agency. All participants provided written informed consent before participation in the study.

### Intervention

The 309 participants in the study sample, aged 62–70 years, were randomly assigned to one of three groups [24]: Heavy Resistance Training (HRT), Moderate Intensity Training (MIT), or a non-exercise control group, as part of a one-year intervention program. Exclusion criteria included significant medical or musculoskeletal conditions, medications likely to interfere with the intervention, and neurological problems [24]. Both exercise groups followed a combined program that included resistance exercises and functional training, designed to improve strength, endurance, and balance. HRT participants attended three supervised sessions per week at a training center. The MIT group attended one supervised session per week and completed two additional unsupervised home-based sessions. Exercise intensity was prescribed individually based on baseline testing and progressed gradually over the intervention period, following established training principles [24]. Adherence in supervised sessions was tracked through attendance logs, and in home-based sessions via participant diaries and regular follow-up calls. The non-exercise group was instructed to maintain their habitual physical activity levels, i.e. less than one hour of strenuous physical activity per week. MRI assessments and exercise performance tests were conducted at baseline, one and two years later. Safety was closely monitored throughout, with all sessions supervised by qualified exercise specialists trained to manage any adverse events. Incidents were promptly documented and reviewed by the study team. MRI assessors and data analysts were blinded to group allocation. No significant demographic differences were found between groups (Table 1). Exercise performance was assessed using isometric leg strength testing of the dominant leg, following standardized protocols [24, 25].

**Table 1:** Demographic characteristics across groups in the study sample

Group	*n*	Age at baseline	Sex (% females)	Years of education
Heavy	103	66.4(2.6)[60.1–70.9]	39	14.2(1.8)[9.5–17]
Moderate	105	66.3(2.5)[60.1–71.0]	39	14.7(2.0)[10.5–17]
non-Exercise	101	66.8(2.4)[62.2–71.0]	41	14.6(7.9)[9.5–17]
Stats	-	F = 1.05, *p* = 0.35	*χ*^2^ = 0.18, *p* = 0.91	F = 1.69, *p* = 0.19

Mean(standard deviation)[range]. Group comparisons for continuous variables were conducted using ANOVA, while binary variables were analyzed with the Chi-square test

### MRI recording and preprocessing

#### Training sample

The training sample had a mean age of 62.3 years (SD = 15.1, range: 31–89), with 46% of the participants being female. Only individuals with both T1-weighted and rs-fMRI data available were included. All participants were cognitively healthy, as indicated by a mean Mini-Mental State Examination (MMSE) score of 30 (SD = 1.8; range = 26–30). Details on the origin of this dataset and a histogram of participants’ ages are provided in Supplementary Material 1. Recordings were obtained under both eyes-open and eyes-closed conditions, which were accounted for as a covariate in subsequent analyses. T1-weighted images were used exclusively for the preprocessing of the functional data. Image quality was verified for all scans through detailed visual inspection. All imaging data were preprocessed using the fMRIPrep (version 22.0.2) pipeline [26] to ensure reproducibility, which includes brain extraction, head motion correction, slice timing correction, co-registration, spatial normalization, field distortion correction, and confound estimation (https://fmriprep.org). Further processing was carried out in Python 3.8 using Nilearn (v0.11.1). Linear regression was used to remove confounding signals from white matter, cerebrospinal fluid, head motion parameters obtained during realignment, and motion-related artifacts (scrubbing). A temporal band-pass filter (0.01–0.1 Hz) was then applied to reduce noise. Time series were extracted for each region in the Automated Anatomical Labeling (AAL) atlas [27]. The resulting data were subsequently harmonized by trimming all time series to 190 volumes and applying z-score normalization, standardizing the data to a mean of 0 and a standard deviation of 1 [28, 29]. From these harmonized time series, pairwise functional connectivity matrices were computed using Pearson correlation. The analysis of the upper triangle of the connectivity matrix yielded 6670 features, which were subsequently used in the analyses.

#### Study sample

All the LISA participants included in the analyses underwent T1-weighted and resting-state fMRI acquisitions on a 3.0 T Philips Achieva scanner (Philips Healthcare) at all time points. T1-weighted images were acquired using a 3D sequence (TR/TE = 6/2.7 ms, flip angle = 8°, matrix size = 288 × 288, 244 slices, isotropic voxel size = 0.853 mm^3^). Resting-state functional images were acquired using a gradient-echo EPI sequence (TR/TE = 2490/30 ms, matrix size = 64 × 64, 42 slices, 240 volumes, isotropic voxel size = 3 × 3 × 3 mm^3^). During the resting-state scan, participants were instructed to keep their eyes open and fixate on a central cross. Image quality was verified for all scans through detailed visual inspection.

The preprocessing and harmonization steps applied to this dataset were identical to those described for the training sample. Preprocessing was conducted at the ROI level, resulting in the extraction of the same set of 6670 features as previously described. Additionally, this sample was also preprocessed at the voxel level employing the CONN toolbox (version 22a) [30]. As part of the CONN preprocessing workflow, images were spatially smoothed using a Gaussian (FWHM = 6 mm). Noise was reduced via linear regression to remove confounding signals from white matter, cerebrospinal fluid, head motion parameters obtained during realignment, and motion-related artifacts (scrubbing). A temporal band-pass filter with a range of 0.01 to 0.1 Hz was applied. Functional connectivity was estimated using the global correlation (GCOR) metric, defined as the mean correlation between each voxel and the rest of the brain [30]. To improve inter-subject comparability of functional data, we applied voxel-wise normalization, transforming the time series of each voxel for each subject into a Gaussian distribution, mean = 0, SD = 1 [30].

### Analysis

#### Voxel-level functional connectivity

First, we examined local connectivity changes between groups, replicating the design of previous studies [9–13]. We conducted a voxel-wise linear mixed-level ANOVA (Group × Time) in CONN to test each possible group comparison for the 1-year contrast relative to baseline. This analysis was based on GCOR [30], controlled for age and sex, and was corrected for multiple comparisons at the cluster level using false discovery rate (FDR), *p* < 0.05.

#### Brain clocks and brain age gaps estimation

Second, we assessed the impact of resistance training on brain aging using brain clock models trained on an independent dataset of 2,433 participants. Brain clock models were implemented in Python (version 3.11.7, 64-bit). A single brain age gap (BAG) value reflects whether the brain appears older or younger than the chronological age at a given time point. The predicted brain age was estimated using Light GBM models trained on 6,670 connectivity-based features. To train the brain clocks, we implemented a machine learning pipeline that included: (1) correction for potential confounding effects of eye-open instructions using a general linear model; (2) feature scaling; (3) univariate feature selection; and (4) model fitting. The brain clock models achieved an R^2^ of 0.405 ± 0.027, an effect size F^2^ of 0.681 ± 0.075, and a MAE of 8.652 ± 0.304 years, demonstrating strong performance consistent with prior work [14–16]. Feature importance was obtained using the split-based importance. The model was repeatedly trained and tested using fivefold cross-validation with 20 repetitions. The estimated BAG was corrected for age-related bias by regressing residuals against chronological age and removing the fitted component [16]. To compare BAGs across the three time points and groups, we employed linear mixed-effects models covarying for age and sex, with both slope and intercept modeled as random factors for each participant (BAGs ∼ time × group + age + sex + (1 + Time∣Subject)). Within each group, paired differences between the 1-year and baseline measurements, as well as between the 2-year and baseline measurements, were assessed using paired permutation (sign-flip) tests. P values across all group/time contrasts were adjusted for multiple testing using the Benjamini–Hochberg FDR. See Supplementary Material 2 for more details on the brain clock models. This entire analysis was first conducted using the complete set of connectivity features, and subsequently repeated for subsets of features restricted to individual resting-state networks (RSNs) to evaluate whether the observed effects were globally distributed or localized within specific functional networks (Supplementary Material 3).

## Results

### Physical training and functional networks

**Fig. 1:**
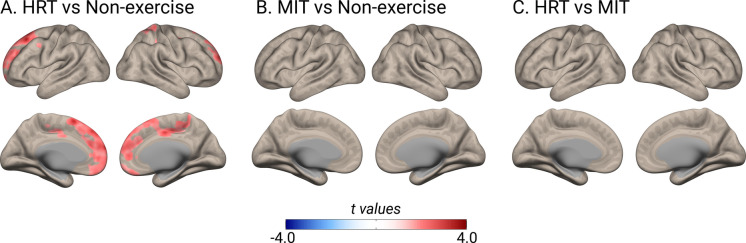
Voxel-level changes in functional connectivity following the physical training intervention. A mixed-effects ANOVA was conducted to evaluate changes from baseline to 1 year (1 year > baseline), testing all pairwise group comparisons (HRT, MIT, and Non-exercise). The analysis was adjusted for age and sex, and multiple comparisons were corrected at the cluster level using the false discovery rate (FDR) method with a threshold of *p* < 0.05. HRT: heavy resistance training, MIT: moderate-intensity resistance training

Voxel-level functional connectivity analysis showed greater activity post-intervention in the HRT group relative to the non-exercise control group (Fig. 1), with a significant cluster (size = 8517 voxels; cluster p_FDR_ < 0.001) primarily encompassing prefrontal regions, with additional involvement of the motor cortex and superior parietal areas (Table 2). No significant differences were observed between the MIT and non-exercise groups, or between the HRT and MIT groups (Fig. 1).

**Table 2:** Anatomical regions included in the significant cluster identified in the functional connectivity analysis

Voxels	% Cluster	% ROI Covered	Region of interest (ROI)	Peak (x, y, z)
1667	20%	24%	Frontal Pole Left	(−14, + 58, + 20)
1114	13%	39%	Superior Frontal Gyrus Left	(−12, + 30, + 50)
930	11%	12%	Frontal Pole Right	(+ 12, + 60, + 24)
408	5%	15%	Superior Frontal Gyrus Right	(+ 8, + 30, + 54)
383	4%	28%	Paracingulate Gyrus Right	(+ 8, + 46, + 18)
324	4%	25%	Paracingulate Gyrus Left	(−8, + 46, + 16)
266	3%	27%	Frontal Medial Cortex	(−2, + 46, −18)
243	3%	9%	Anterior Cingulate Gyrus	(+ 2, + 0, + 38)
160	2%	5%	Postcentral Gyrus Right	(+ 16, −40, + 64)
143	2%	10%	Superior Parietal Lobule Right	(+ 26, −50, + 68)
132	2%	3%	Precentral Gyrus Right	(+ 8, −26, + 50)
78	1%	11%	Supplementary Motor Cortex Right	(+ 12, −2, + 48)
71	1%	2%	Middle Frontal Gyrus Left	(−28, + 18, + 46)
50	1%	8%	Supplementary Motor Cortex Left	(−8, −8, + 48)

### Physical training and brain aging

#### Whole brain

**Fig. 2:**
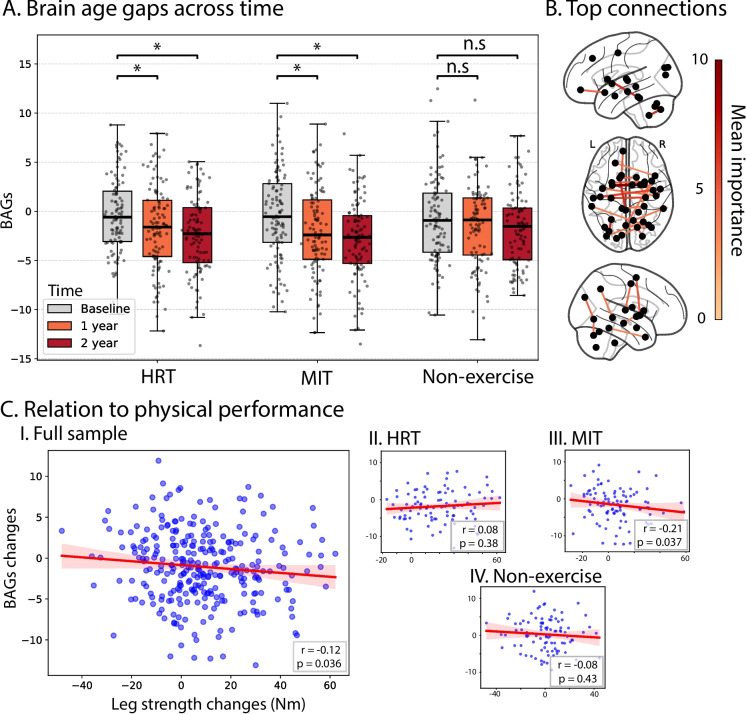
Changes in brain age gap (BAG) over time across exercise groups. **A **Plot showing BAG differences across time points and exercise groups. BAG comparisons were conducted through paired permutation tests, with multiple-comparison correction using the False Discovery Rate (FDR). **B **Top connections identified by the brain clock models contributing to BAG predictions. **C** Association between changes in BAG and changes in leg strength. n.s.: non-significant, *: *p*_FDR_ < 0.05. C. HRT: heavy resistance training, MIT: moderate-intensity resistance training.. A. Brain age gaps across time. B. Top connections. n.s. n.s. *. *. *. 10. *. 5. 0. Time. HRT. Non-exercise. MIT. C. Relation to physical performance. I. Full sample. II. HRT. III. MIT. 10. 10. 0. 0. *r* = −0.21. *p* = 0.037. *r* = 0.08. *p* = 0.38. −10. −10. −20. 0. 60. −20. 0. 60. IV. Non-exercise. 10. 0. *r* = −0.12. *p* = 0.036. *r* = −0.08. *p* = 0.43. 40. −10. −40. 0. Leg strength changes (Nm)

BAGs decreased post-intervention only in the exercise groups, whereas no significant change was observed in the non-exercise control group (Fig. 2A). The brain clock model engaged widespread networks, including frontal, temporal, subcortical, and cerebellar connections (Fig. 2B, Supplementary Material 4)**.** The linear mixed model shows a significant interaction between time and group (Table 3). Subsequent permutation comparisons (Table 4) showed a reduction in BAG of 1.4 years at 1 year and 1.84 years at 2 years relative to baseline in the HRT group. Similarly, in the MIT group, the reductions were 1.39 and 2.26 years, respectively. No significant changes were observed in the non-exercise group. There was a significant Spearman correlation between changes in BAG from baseline to the 1-year follow-up and changes in leg strength (r = −0.12,* p* = 0.036, Fig. 2C). When analyzed by group, this association was not significant in the heavy (r = 0.09, *p *= 0.384) or non-exercise group (r = −0.08, *p* = 0.427) but reached significance in the moderate group (r = −0.21, *p *= 0.038), Fig. 2C. Individual variability was analyzed in Supplementary Material 5.

**Table 3:** Fixed effects in the linear mixed-effects model

Term	k	χ^2^	df	*p* value	η^2^p
Age (baseline)	1	30.92	1	< 0.001	0.049
Sex	1	1.96	1	0.162	0.003
Time	2	4.22	2	0.127	0.007
Group	2	0.10	2	0.947	0.000
Time × Group	4	10.21	4	0.031	0.017

Df: degree of freedom, k: number of fixed coefficients, η^2^*p*: partial eta squared, χ^2^: chi square

**Table 4:** Group-wise paired permutations test of BAG between baseline and follow-up

Group	Time comparison	Mean difference	p_unc_	Cohen's d (paired)	N	p_FDR_
HRT	1 year—baseline	−1.42	0.002	−0.38	74	0.003
HRT	2 year—baseline	−1.85	< 0.001	−0.52	100	< 0.001
MIT	1 year—baseline	−1.39	0.007	−0.32	75	0.010
MIT	2 year—baseline	−2.26	< 0.001	−0.51	102	< 0.001
Non-exercise	1 year—baseline	−0.66	0.218	−0.15	73	0.218
Non-exercise	2 year—baseline	−0.82	0.181	−0.18	97	0.197

*FDR* false discovery correction,* unc* uncorrected

#### Specific RSN

Restricting the brain clocks models to specific RSN did not yield any statistically significant effects in the interaction time x group (default mode network: χ^2^ = 1.95, *p* = 0.38, motor network: χ^2^ = 1.37, *p* = 0.69, somatosensory network: χ^2^ = 1.82, *p* = 0.44, cerebellar network: χ^2^ = 1.56, *p* = 0.21, auditory network: χ^2^ = 1.94, *p* = 0.11 and visual network: χ^2^ = 1.50, *p* = 0.22); (Supplementary Material 3).

## Discussion

This study used rs-fMRI to investigate the impact of one year of resistance training on brain aging in older adults. Our results show that both heavy and moderate resistance training slowed brain aging, as estimated by brain clock models, whereas no effect was observed in the control group. Exercise-related changes in functional connectivity [31] extended beyond specific brain regions, reflecting global improvements in brain health. These findings highlight resistance training as a key modifiable lifestyle factor [1, 4, 31–33] capable of delaying brain aging and supporting healthy brain function later in life [34, 35].

Our analyses revealed evidence for a training-related delay in brain aging, reflecting widespread functional changes rather than effects limited to specific networks such as the default mode, motor, or cerebellar systems. The results suggest a hierarchical organization of the impact on brain aging, driven by distributed network-level changes and expressed through regional focal patterns revealed by voxel-wise analyses of functional connectivity. The increased prefrontal connectivity observed in the HRT group indicates localized enhancements within a globally coordinated reorganization. As the prefrontal cortex supports attention, executive control, and working memory [36, 37], its strengthened connectivity may represent a mechanistic link between exercise and cognitive improvements reported after high-intensity training [23]. While previous studies have highlighted isolated regional effects in the hippocampus, motor, and prefrontal cortices [14–16], they could not determine whether such changes were local or part of a whole-brain adaptation. Our findings support the latter, aligning with evidence that exercise promotes plasticity through synaptic, angiogenic, neurotrophic, and vascular mechanisms [1, 31, 32, 38]. The prefrontal cortex likely reflects the local expression of this global phenomenon, where cognitive engagement during exercise further reinforces executive networks [37, 39, 40].

In the LISA study, both strength training groups (HRT and MIT) increased leg strength after one year and maintained these gains a year later [25]. In parallel, the modest reduction in brain age gap (~ 1–2 years) is consistent with effect sizes reported in prior brain age studies of lifestyle and health factors, including physical activity, education, and cardiometabolic risk [21, 41–43]. Given that brain aging is a gradual and cumulative process, differences of this magnitude are considered biologically meaningful and have been linked to improved brain integrity and cognitive performance in older adults [43–46]. From a clinical perspective, these findings suggest that regular resistance exercise may support more favorable brain aging trajectories and help maintain cognitive capacities over time. In this context, the absence of significant effects in network-specific (RSN-based) brain clock analyses may partly reflect limited sensitivity of current RSN-level models to detect subtle intervention-related changes. However, given the widespread connectivity alterations observed in our voxel-wise analyses, we consider it more plausible that resistance training influences brain aging primarily through global, distributed mechanisms rather than isolated network-specific effects. Such global effects are likely driven by systemic molecular and vascular processes induced by exercise, as described above. Moreover, these subnetworks, while partially overlapping with the brain age network, are not optimized for age prediction and may therefore be less sensitive to detecting BAG differences. In our analysis, we found a modest association between improvements in physical performance and reduced BAGs only in the MIT group. This pattern fits the expected group dynamics: HRT likely produced broad benefits across participants, the control group showed none, and MIT benefits appeared in only some individuals. The absence of a BAG–performance association in HRT may reflect a ceiling effect. This interpretation aligns with evidence of a non-linear dose–response relationship between exercise and BAG reduction [47], in which greater training exposure does not necessarily yield proportionally greater brain benefits. The “weekend warrior” effect—short but intense weekly exercise—illustrates that substantial brain and health benefits can emerge even in the absence of linear performance scaling [48, 49]. In addition, baseline fitness levels, individual responsiveness to training, and potential measurement noise in both physical performance and neuroimaging-derived BAG estimates may further contribute to the observed pattern [10, 50–52]. Together, these factors help reconcile the modest and group-specific performance–BAG associations with the broader, globally distributed mechanisms of exercise-related brain aging proposed in this study.

Our study has several strengths and significant clinical implications. The randomized controlled design provides strong methodological rigor. The one-year supervised intervention allowed us to assess the longitudinal impact of resistance training on brain aging, overcoming limitations of previous cross-sectional work [11–13]. Brain age was estimated using externally trained models (n = 2,433), ensuring generalizability and minimizing bias [14–16]. The use of high-dimensional functional connectivity data (6,670 features) within a reproducible, harmonized pipeline further supports the robustness of our findings. The benefits, lasting one year after the intervention and not observed in the non-exercise group, demonstrate that resistance training has lasting effects beyond the training period. By linking exercise performance with these computational markers of brain aging, our study connects clinical outcomes with modern modeling tools, highlighting the potential of brain clocks to track and personalize lifestyle interventions.

Despite these strengths, several limitations warrant consideration. The generalizability of our findings is limited to healthy, older adults from a high-income European context, which restricts their applicability to more diverse populations, including those with comorbidities or from low- and middle-income countries. While reductions in BAG were statistically significant (1–2 years), their clinical significance should be tested in longer-term follow-up. Network-specific brain clocks did not reveal significant differences, suggesting that current models may lack sensitivity to detect subsystem-level effects, or that exercise exerts its influence primarily through global rather than localized mechanisms. Finally, associations with muscle strength were relatively small and inconsistent across groups, suggesting that other behavioral or biological mediators may better explain the observed benefits of brain aging.

In conclusion, resistance training slows down brain aging and supports its use as a preventive strategy for maintaining brain health. Both training intensities reduced brain age gaps, suggesting that even moderate exercise can yield measurable benefits. These results highlight the public health benefits of structured exercise programs and the utility of brain clocks as biomarkers to monitor the effects of lifestyle interventions. Future studies should test these effects in more diverse populations and explore additional mechanisms underlying exercise-related brain improvements.

## Supplementary Information

Below is the link to the electronic supplementary material.

ESM 1 (DOCX 197 KB)

## Data Availability

Data are available upon reasonable request, subject to approval for ethical and data protection purposes, by contacting the corresponding authors.
