# Supplementary material for: Randomized controlled trial of resistance exercise and brain aging clocks
Source: GeroScience. 2026 Feb 10;48(3):3251–62. doi: 10.1007/s11357-026-02141-x (PMC13356001; doi:10.1007/s11357-026-02141-x)
Supplement: Supplementary file 1 — (DOCX 197 KB) [file 11357_2026_2141_MOESM1_ESM.docx]

**Supplementary Material 1.** Data origin of training sample

## Detailed information about the datasets used in the external rs-fMRI cohort, including their origin, publication references, data access procedures, and sample sizes, is provided in **Supplementary Table S1**. In addition, **Supplementary Figures 1 and 2 display histograms showing the age distributions** of the participants included in the external datasets and intervention group, respectively.

**Supplementary Table S1.** External rs-fMRI dataset details

| Dataset | Country | Access | n(HCs) |  |
| --- | --- | --- | --- | --- |
| ADNI [1] | USA | <https://ida.loni.usc.edu/> | 468 |  |
| Cam-Can [2] | United Kingdom | <https://camcan-archive.mrc-cbu.cam.ac.uk/> | 576 |  |
| NIFD [3] | USA | <https://ida.loni.usc.edu/> | 108 |  |
| HCP [4] | USA | <https://www.humanconnectome.org/> | 688 |  |
| Lemon [5] | Germany | <https://fcon_1000.projects.nitrc.org/> | 87 |  |
| ReDLat [6] | Argentina, Brazil, Colombia, Chile, Mexico, Peru, USA | <https://red-lat.com/> | 506 |  |


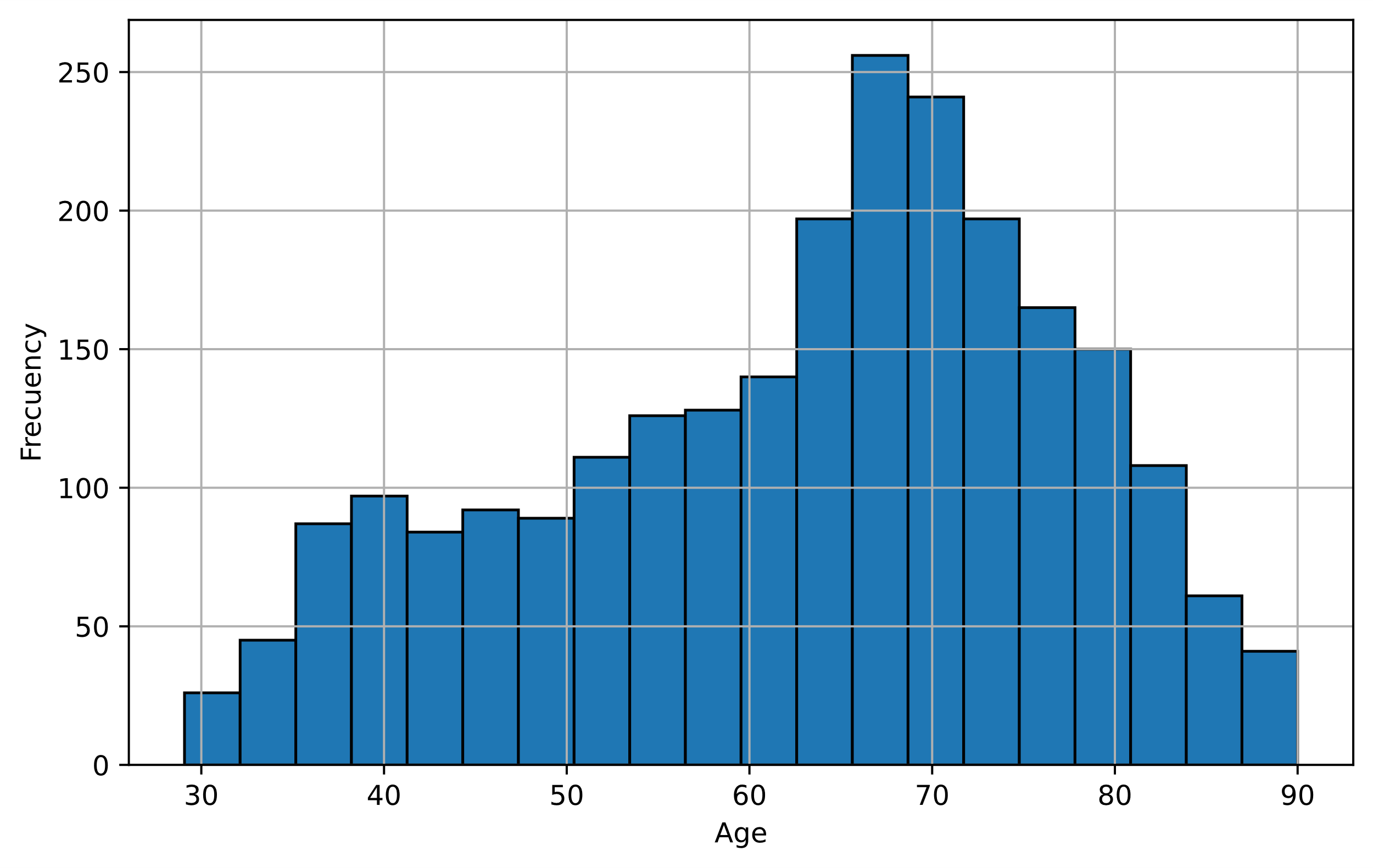


**Supplementary Figure 1.** Age distribution of participants included in the external rs-fMRI datasets


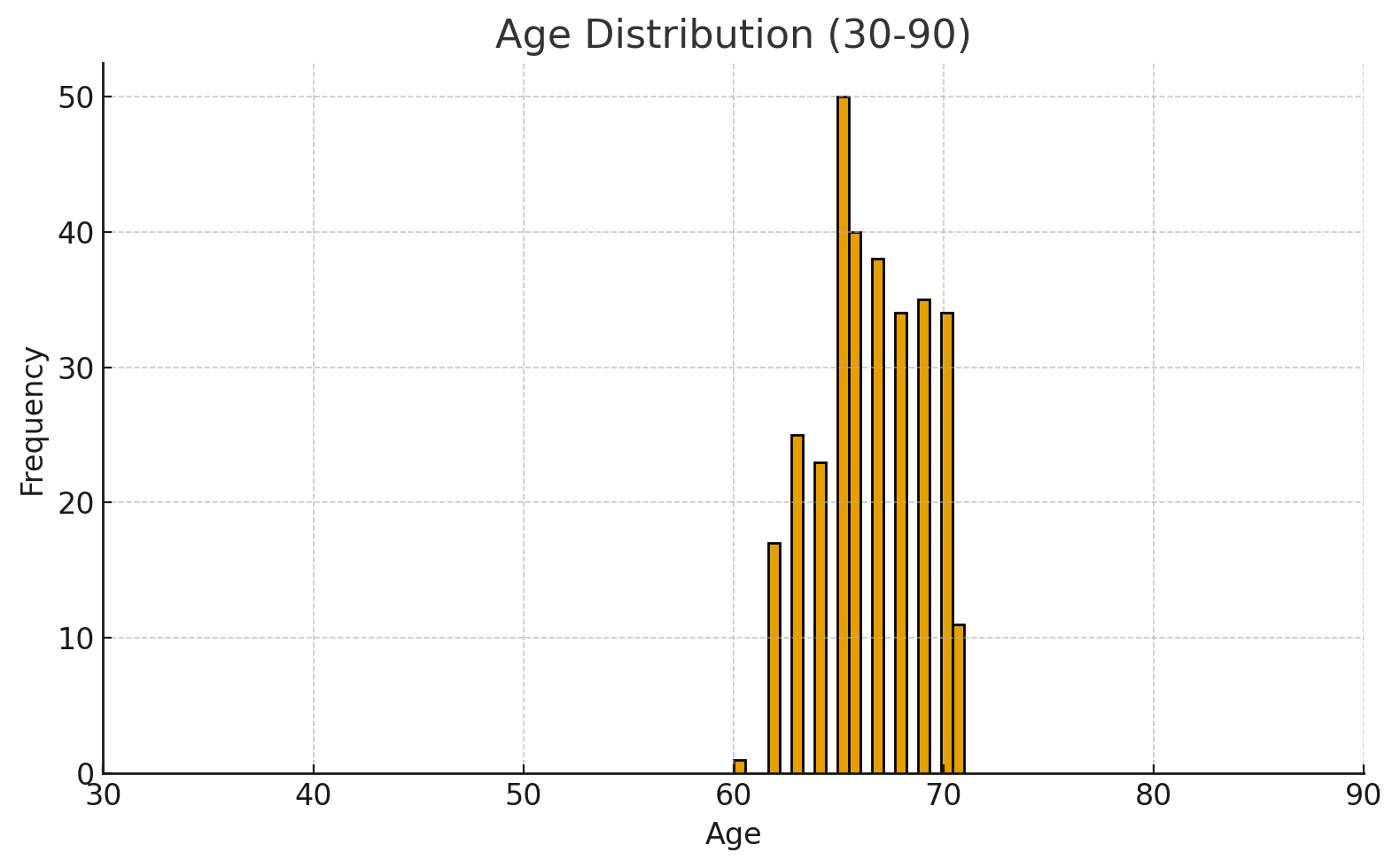


**Supplementary Figure 2.** Age distribution of participants included in the intervention dataset.

**Supplementary Material 2. Details of brain clock models**

All connectivity-based features were first corrected for eye condition (open vs. closed) using a general linear model (GLM) to minimize potential confounding effects, with the resulting residuals used as input for the machine learning pipeline. This pipeline, implemented using LightGBM, included min–max feature scaling (range 0.05–0.95) fitted exclusively on the training data within each fold and subsequently applied—without re-fitting—to the corresponding test data and to the study sample, ensuring consistency and preventing data leakage. This model employs an ensemble of decision trees optimized via gradient boosting, which sequentially improves prediction accuracy by focusing on the residual errors of previous trees. Model performance was evaluated using the coefficient of determination (R²), mean absolute error (MAE), and F² scores.

The pipeline also included univariate feature selection using *f*-regression to retain the most informative features and model fitting with gradient boosting decision trees. Hyperparameter optimization was conducted using Bayesian search to efficiently explore the parameter space while balancing exploration and exploitation, performed within an inner 5-fold cross-validation loop nested inside an outer 5×5 repeated K-fold cross-validation, with mean absolute error (MAE) as the optimization metric. The most frequently occurring hyperparameter configuration across the outer folds was selected for the final model, which was then trained and tested using 5-fold cross-validation repeated 20 times. In each fold and repetition, the study sample was tested separately for each group and time point, and performance metrics and feature importance values were subsequently averaged across all folds and repetitions. BAG was calculated as the difference between predicted and chronological age and was corrected for age-related bias by regressing residuals against chronological age and subtracting the fitted component, yielding a bias-adjusted BAG for each subject, including those in the held-out test set and each LISA group at both time points. Analyses were performed both using the complete set of connectivity features (i.e., all brain connections) and with features restricted to RSNs to assess whether the observed effects were globally distributed or localized within particular functional networks. Model performance was evaluated using the coefficient of determination (R²), MAE, and F² scores, while feature importance was computed within each fold and repetition and averaged to produce a final ranking of the relative contribution of each feature.

**Supplementary Material 3. Network-specific brain clocks**

We developed network-specific brain clocks to estimate brain age using only the functional connectivity within predefined large-scale networks. For each network, we selected bilateral core ROIs that are consistently associated with the corresponding functional system. The default mode network included the frontal superior medial, posterior cingulate, and angular gyri. The motor network comprised the precentral gyrus, supplementary motor area, and thalamus. For the somatosensory network, we selected the postcentral gyrus, superior parietal lobule, and paracentral lobule. The cerebellar network included lobules 4/5, 6, and 8 of the cerebellum, as well as vermis regions 1/2, 3, 4/5, and 6. The visual network consisted of the calcarine cortex, cuneus, lingual gyrus, and superior occipital gyrus. Lastly, the auditory network included Heschl’s gyrus, the superior temporal gyrus, and the middle temporal gyrus. For each network, models were trained using only the functional connectivity between all brain ROIs and the selected ROIs belonging to that specific network. This approach allowed us to isolate the contribution of each functional system to brain age prediction while maintaining whole-brain contextual information. The performance of each network-specific model is presented in **Supplementary Table 2**.

**Supplementary Table 2.** Performance of network-specific brain clocks using Pearson correlation as the connectivity measure.

| **Network** | **R² (Mean ± SD)** | **F^2^ (Mean ± SD)** | **MAE (Mean ± SD)** |
| --- | --- | --- | --- |
| Default mode | 0.34 ± 0.03 | 0.51 ± 0.07 | 11.87 ± 0.38 |
| Motor | 0.37 ± 0.03 | 0.60 ± 0.07 | 11.47 ± 0.31 |
| Somatosensory | 0.32 ± 0.03 | 0.46 ± 0.07 | 12.08 ± 0.35 |
| Cerebellar | 0.33 ± 0.03 | 0.49 ± 0.06 | 11.89 ± 0.34 |
| Auditory | 0.35 ± 0.03 | 0.53 ± 0.08 | 11.73 ± 0.38 |
| Visual | 0.29 ± 0.03 | 0.41 ± 0.06 | 12.35 ± 0.35 |

**Supplementary Material 4. Feature Importance of Pearson-Based Brain Clock**

**Supplementary Table 3** presents the mean and standard deviation of the importance values for the top 50 connections used in the Pearson-based brain clock models, calculated across 5-fold cross-validation and 20 repetitions.

**Supplementary Table 3.** Importance scores of the top 50 functional connections in Pearson-based brain clock models.

| **ROI1** | **ROI2** | **Mean Importance** | **Std Importance** |
| --- | --- | --- | --- |
| Heschl L | Temporal Sup L | 10.50 | 2.26 |
| Amygdala L | Amygdala R | 10.38 | 2.80 |
| Putamen R | Pallidum R | 9.98 | 2.87 |
| Cerebelum Crus2 L | Cerebelum 8 L | 6.88 | 1.48 |
| Caudate L | Vermis 1 2 | 6.83 | 2.50 |
| Rolandic Oper R | Heschl L | 6.12 | 2.07 |
| Hippocampus L | Hippocampus R | 6.00 | 2.04 |
| Rolandic Oper R | Heschl R | 5.66 | 1.95 |
| Supp Motor Area R | Insula R | 5.25 | 1.39 |
| Caudate L | Caudate R | 5.12 | 1.92 |
| Olfactory L | Frontal Med Orb L | 5.03 | 2.04 |
| Lingual R | Occipital Sup R | 4.88 | 2.05 |
| Precentral R | Heschl R | 4.74 | 1.67 |
| Putamen L | Putamen R | 4.72 | 1.92 |
| Lingual R | Cerebelum Crus1 R | 4.69 | 1.74 |
| Rolandic Oper R | Temporal Sup L | 4.45 | 1.99 |
| Insula R | Vermis 8 | 4.36 | 1.78 |
| Cerebelum 3 L | Cerebelum 10 R | 4.33 | 2.11 |
| Cerebelum Crus1 L | Cerebelum Crus2 L | 4.27 | 2.63 |
| Precentral L | Heschl R | 4.22 | 2.17 |
| Cerebelum 7b R | Vermis 8 | 4.15 | 1.63 |
| Occipital Mid L | Cerebelum 6 R | 4.04 | 1.88 |
| Precuneus R | Temporal Mid R | 4.02 | 2.09 |
| Lingual R | Occipital Sup L | 3.98 | 2.12 |
| Temporal Mid L | Cerebelum Crus1 R | 3.78 | 1.97 |
| Hippocampus R | Caudate R | 3.77 | 2.21 |
| Precentral R | Heschl L | 3.54 | 1.38 |
| Temporal Pol Mid R | Cerebelum 4 5 R | 3.52 | 1.77 |
| Frontal Sup L | Temporal Pol Mid R | 3.44 | 1.72 |
| Cuneus L | Occipital Sup L | 3.43 | 1.94 |
| Frontal Sup L | Frontal Mid R | 3.39 | 1.41 |
| ParaHippocampal R | Temporal Pol Mid R | 3.39 | 1.88 |
| Supp Motor Area R | Paracentral Lob L | 3.34 | 2.04 |
| Cingulum Post R | Cerebelum 4 5 L | 3.32 | 2.16 |
| Angular R | Precuneus R | 3.30 | 2.04 |
| Precentral R | Supp Motor Area R | 3.19 | 1.92 |
| Precuneus R | Vermis 9 | 3.09 | 1.81 |
| Frontal Inf Oper R | Insula R | 3.04 | 2.07 |
| Frontal Sup Med R | Temporal Pol Mid R | 3.03 | 1.76 |
| Pallidum R | Cerebelum 9 L | 2.99 | 1.97 |
| Frontal Sup Med L | Temporal Pol Mid R | 2.98 | 1.61 |
| Frontal Mid Orb R | Cerebelum 8 L | 2.93 | 1.87 |
| Cingulum Post L | Putamen L | 2.91 | 1.91 |
| Temporal Inf L | Cerebelum Crus1 R | 2.91 | 2.02 |
| Temporal Pole Sup R | Temporal Mid R | 2.87 | 1.68 |
| Cingulum Ant L | Vermis 7 | 2.85 | 1.67 |
| Amygdala R | Cerebelum 9 R | 2.84 | 1.68 |
| Frontal Sup L | Temporal Pole Sup L | 2.84 | 1.75 |
| ParaHippocampal R | Putamen L | 2.82 | 2.09 |
| Insula L | Paracentral Lob L | 2.73 | 1.79 |
| Frontal Med Orb L | Frontal Med Orb R | 2.72 | 1.92 |

**Supplementary Material 5. Individual variability**

To further explore the robustness of our findings, we assessed whether individual variability within each group was larger than the between-group differences in BAG. Brown–Forsythe tests of homogeneity of variance showed no significant differences in variability across groups at baseline, 1-year, or 2-year follow-ups (all p > 0.35). Consistently, effect size indices (η² range = 0.001–0.018; ω² range = –0.005–0.012) indicated that between-group variability accounted for less than 2% of the total variance. The ratio of between- to within-group variance was very low across all time points (0.2–2%) and remained negligible when pooling all time points together (0.5%). These results confirm that intra-group variability vastly outweighed inter-group differences, highlighting substantial individual heterogeneity in BAG trajectories.

**References**

1. Petersen, R.C., et al., *Alzheimer's Disease Neuroimaging Initiative (ADNI): Clinical characterization.* Neurology, 2010. **74**: p. 201-209.

2. Shafto, M.A., et al., *The Cambridge Centre for Ageing and Neuroscience (Cam-CAN) study protocol: a cross-sectional, lifespan, multidisciplinary examination of healthy cognitive ageing.* BMC Neurology, 2014. **14**(1): p. 204.

3. Rohrer, J.D. and H.J. Rosen, *Neuroimaging in frontotemporal dementia.* International Review of Psychiatry, 2013. **25**: p. 221-229.

4. Bookheimer, S.Y., et al., *The Lifespan Human Connectome Project in Aging: An overview.* Neuroimage, 2019. **185**: p. 335-348.

5. Babayan, A., et al., *A mind-brain-body dataset of MRI, EEG, cognition, emotion, and peripheral physiology in young and old adults.* Sci Data, 2019. **6**: p. 180308.

6. Ibanez, A., et al., *The Multi-Partner Consortium to Expand Dementia Research in Latin America (ReDLat): Driving Multicentric Research and Implementation Science*, in *Frontiers in Neurology*. 2021, Front Neurol. p. 631722.
